# (9*R*,10*R*,10a*R*)-9-(2-Bromo­phen­yl)-10-nitro-6-phenyl-10,10a-dihydro-9*H*-benzo[*c*]chromene-8-carbaldehyde

**DOI:** 10.1107/S160053681103995X

**Published:** 2011-10-05

**Authors:** Jeremy Dufour, Modhu Sudan Maji, Michael Bolte

**Affiliations:** a Institute of Organic Chemistry, RWTH Aachen University, Landoltweg 1, 52074 Aachen, Germany; b Institut für Anorganische Chemie, J. W. Goethe-Universität Frankfurt, Max-von-Laue-Strasse 7, 60438 Frankfurt/Main, Germany

## Abstract

The title compound, C_26_H_18_BrNO_4_, features a functionalized chromene. The cyclo­hexene ring adopts a sofa conformation and has the nitro group and the bromo­phenyl ring in an axial position. The ten atoms of the chromene moiety lie close to a common plane (r.m.s. deviation = 0.066 Å). The attached phenyl ring is twisted by 32.89 (10)° from the chromene plane. The crystal packing is stabilized by C—H⋯O inter­actions.

## Related literature

For functionalized chromenes, see: Ellis & Lockhart (2007 ▶). For the synthesis of the title compound, see: Rueping *et al.* (2011 ▶).
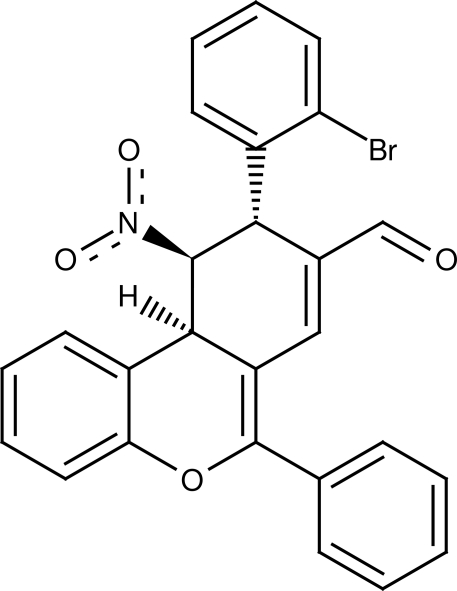


## Experimental

### 

#### Crystal data

C_26_H_18_BrNO_4_
*M* *_r_* = 488.32
Monoclinic, 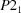

*a* = 7.1583 (8) Å
*b* = 13.2036 (12) Å
*c* = 11.2510 (14) Å
β = 90.642 (9)°
*V* = 1063.3 (2) Å^3^
*Z* = 2
Mo *K*α radiation
μ = 1.97 mm^−1^
*T* = 173 K
0.36 × 0.35 × 0.19 mm

#### Data collection

Stoe IPDS II two-circle diffractometer
Absorption correction: multi-scan (*MULABS*; Spek, 2009 ▶; Blessing, 1995 ▶) *T*_min_ = 0.538, *T*_max_ = 0.706
6069 measured reflections
3778 independent reflections
3443 reflections with *I* > 2σ(*I*)
*R*_int_ = 0.034

#### Refinement

*R*[*F* ^2^ > 2σ(*F* ^2^)] = 0.036
*wR*(*F* ^2^) = 0.087
*S* = 1.00
3778 reflections
290 parameters
1 restraint
H-atom parameters constrained
Δρ_max_ = 0.59 e Å^−3^
Δρ_min_ = −0.60 e Å^−3^
Absolute structure: Flack (1983 ▶), 1700 Friedel pairs
Flack parameter: −0.003 (8)

### 

Data collection: *X-AREA* (Stoe & Cie, 2001 ▶); cell refinement: *X-AREA*; data reduction: *X-AREA*; program(s) used to solve structure: *SHELXS97* (Sheldrick, 2008 ▶); program(s) used to refine structure: *SHELXL97* (Sheldrick, 2008 ▶); molecular graphics: *XP* (Sheldrick, 2008 ▶); software used to prepare material for publication: *SHELXL97*.

## Supplementary Material

Crystal structure: contains datablock(s) I, global. DOI: 10.1107/S160053681103995X/qm2032sup1.cif

Structure factors: contains datablock(s) I. DOI: 10.1107/S160053681103995X/qm2032Isup2.hkl

Supplementary material file. DOI: 10.1107/S160053681103995X/qm2032Isup3.cml

Additional supplementary materials:  crystallographic information; 3D view; checkCIF report

## Figures and Tables

**Table 1:** Hydrogen-bond geometry (Å, °)

*D*—H⋯*A*	*D*—H	H⋯*A*	*D*⋯*A*	*D*—H⋯*A*
C4—H4⋯O15^i^	1.00	2.34	3.266 (4)	154
C7—H7⋯O15^ii^	0.95	2.55	3.487 (5)	168
C25—H25⋯O15^iii^	0.95	2.45	3.314 (4)	151

Symmetry codes: (i) 
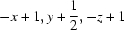
; (ii) 
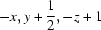
; (iii) 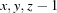.

**Fig. 1.**
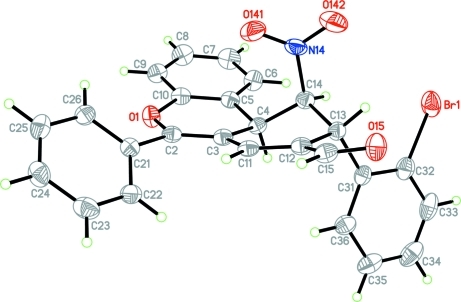
Perspective view of the title compound with the atom numbering; displacement ellipsoids are at the 50% probability level.

## supplementary crystallographic information

### Comment

Functionalized chromenes are important compounds due to their biological activity. They find wide application in medicinal chemistry and have shown to display spasmolytic, diuretic, clotting, antiviral, anti-tumoral and anti-anaphylactic activity. Additionally, they can be used as pigments, photo-active materials and biodegradable agrochemicals. Furthermore, chromenes are components of numerous natural products (Ellis & Lockhart, 2007). The title compound was prepared following two new consecutive domino reactions starting from readily available propargylic alcohols, nitro-styrenes and α,β-unsaturated aldehydes.

The title compound features a functionalized chromene. The cyclohexene ring adopts a sofa conformation and has the nitro group and the bromophenyl ring in an axial position. The ten atoms of the chromene moiety lie in a common plane (r.m.s. deviation 0.066 Å). The attached phenyl ring is twisted by 32.89 (10)° from the chromene plane. The crystal packing is stabilized by C—H···O interactions.

### Experimental

The title compound has been synthesized as described by Rueping *et al.* (2011).

### Refinement

All H atoms could be located by difference Fourier synthesis. They were refined with fixed individual displacement parameters [U(H) = 1.2 *U*_eq_(C)] using a riding model with C—H ranging from 0.95Å to 1.00 Å.

### Crystal data

**Table:**

C_26_H_18_BrNO_4_	*F*(000) = 496
*M**_r_* = 488.32	*D*_x_ = 1.525 Mg m^−^^3^
Monoclinic, *P*2_1_	Mo *K*α radiation, λ = 0.71073 Å
Hall symbol: P 2yb	Cell parameters from 5380 reflections
*a* = 7.1583 (8) Å	θ = 3.6–25.9°
*b* = 13.2036 (12) Å	µ = 1.97 mm^−^^1^
*c* = 11.2510 (14) Å	*T* = 173 K
β = 90.642 (9)°	Plate, yellow
*V* = 1063.3 (2) Å^3^	0.36 × 0.35 × 0.19 mm
*Z* = 2	

### Data collection

**Table:**

Stoe IPDS II two-circle diffractometer	3778 independent reflections
Radiation source: fine-focus sealed tube	3443 reflections with *I* > 2σ(*I*)
graphite	*R*_int_ = 0.034
ω scans	θ_max_ = 25.6°, θ_min_ = 3.6°
Absorption correction: multi-scan (*MULABS*; Spek, 2009; Blessing, 1995)	*h* = −8→8
*T*_min_ = 0.538, *T*_max_ = 0.706	*k* = −16→16
6069 measured reflections	*l* = −11→13

### Refinement

**Table:**

Refinement on *F*^2^	Hydrogen site location: inferred from neighbouring sites
Least-squares matrix: full	H-atom parameters constrained
*R*[*F*^2^ > 2σ(*F*^2^)] = 0.036	*w* = 1/[σ^2^(*F*_o_^2^) + (0.0608*P*)^2^] where *P* = (*F*_o_^2^ + 2*F*_c_^2^)/3
*wR*(*F*^2^) = 0.087	(Δ/σ)_max_ = 0.001
*S* = 1.00	Δρ_max_ = 0.59 e Å^−^^3^
3778 reflections	Δρ_min_ = −0.60 e Å^−^^3^
290 parameters	Extinction correction: *SHELXL97* (Sheldrick, 2008), Fc^*^=kFc[1+0.001xFc^2^λ^3^/sin(2θ)]^-1/4^
1 restraint	Extinction coefficient: 0.021 (2)
Primary atom site location: structure-invariant direct methods	Absolute structure: Flack (1983), 1700 Friedel pairs
Secondary atom site location: difference Fourier map	Flack parameter: −0.003 (8)

### Special details

**Table:**

Experimental. ;
Geometry. All e.s.d.'s (except the e.s.d. in the dihedral angle between two l.s. planes) are estimated using the full covariance matrix. The cell e.s.d.'s are taken into account individually in the estimation of e.s.d.'s in distances, angles and torsion angles; correlations between e.s.d.'s in cell parameters are only used when they are defined by crystal symmetry. An approximate (isotropic) treatment of cell e.s.d.'s is used for estimating e.s.d.'s involving l.s. planes.
Refinement. Refinement of *F*^2^ against ALL reflections. The weighted *R*-factor *wR* and goodness of fit *S* are based on *F*^2^, conventional *R*-factors *R* are based on *F*, with *F* set to zero for negative *F*^2^. The threshold expression of *F*^2^ > σ(*F*^2^) is used only for calculating *R*-factors(gt) *etc*. and is not relevant to the choice of reflections for refinement. *R*-factors based on *F*^2^ are statistically about twice as large as those based on *F*, and *R*- factors based on ALL data will be even larger.

### Fractional atomic coordinates and isotropic or equivalent isotropic displacement parameters (Å^2^)

**Table:**

	*x*	*y*	*z*	*U*_iso_*/*U*_eq_	
Br1	0.10708 (4)	0.57378 (3)	0.75261 (3)	0.04064 (14)	
O1	0.2792 (3)	0.61771 (19)	0.0890 (2)	0.0276 (5)	
C2	0.4038 (4)	0.5584 (2)	0.1524 (3)	0.0227 (6)	
C3	0.3955 (4)	0.5501 (2)	0.2723 (3)	0.0231 (7)	
C4	0.2625 (4)	0.6123 (2)	0.3462 (3)	0.0222 (6)	
H4	0.3374	0.6693	0.3808	0.027*	
C5	0.1122 (4)	0.6604 (2)	0.2682 (3)	0.0240 (7)	
C6	−0.0442 (5)	0.7082 (3)	0.3160 (4)	0.0295 (8)	
H6	−0.0573	0.7119	0.3998	0.035*	
C7	−0.1813 (5)	0.7506 (3)	0.2423 (4)	0.0360 (8)	
H7	−0.2868	0.7830	0.2760	0.043*	
C8	−0.1636 (5)	0.7455 (3)	0.1208 (4)	0.0348 (8)	
H8	−0.2588	0.7729	0.0709	0.042*	
C9	−0.0093 (5)	0.7010 (3)	0.0707 (3)	0.0299 (7)	
H9	0.0040	0.6986	−0.0131	0.036*	
C10	0.1272 (5)	0.6595 (2)	0.1459 (3)	0.0246 (7)	
C11	0.5043 (4)	0.4747 (2)	0.3365 (3)	0.0227 (6)	
H11	0.5940	0.4370	0.2934	0.027*	
C12	0.4863 (4)	0.4550 (2)	0.4530 (3)	0.0237 (7)	
C13	0.3483 (4)	0.5097 (3)	0.5309 (3)	0.0234 (7)	
H13	0.2916	0.4581	0.5847	0.028*	
C14	0.1891 (4)	0.5519 (2)	0.4502 (3)	0.0251 (7)	
H14	0.1088	0.5973	0.4991	0.030*	
N14	0.0711 (4)	0.4609 (3)	0.4096 (3)	0.0340 (7)	
O141	0.0475 (4)	0.4442 (2)	0.3057 (3)	0.0426 (7)	
O142	0.0106 (6)	0.4048 (4)	0.4866 (4)	0.0808 (14)	
C15	0.5881 (5)	0.3690 (2)	0.5044 (3)	0.0261 (7)	
H15	0.6739	0.3343	0.4551	0.031*	
O15	0.5706 (4)	0.33907 (19)	0.6056 (2)	0.0329 (6)	
C21	0.5311 (4)	0.5078 (2)	0.0706 (3)	0.0233 (7)	
C22	0.7220 (5)	0.4978 (3)	0.0954 (3)	0.0286 (7)	
H22	0.7722	0.5223	0.1684	0.034*	
C23	0.8374 (5)	0.4526 (3)	0.0140 (4)	0.0368 (8)	
H23	0.9667	0.4448	0.0318	0.044*	
C24	0.7662 (5)	0.4180 (3)	−0.0946 (3)	0.0341 (8)	
H24	0.8465	0.3878	−0.1511	0.041*	
C25	0.5773 (5)	0.4282 (3)	−0.1190 (3)	0.0333 (8)	
H25	0.5278	0.4044	−0.1926	0.040*	
C26	0.4612 (5)	0.4722 (3)	−0.0384 (3)	0.0283 (7)	
H26	0.3319	0.4788	−0.0565	0.034*	
C31	0.4333 (4)	0.5917 (2)	0.6090 (3)	0.0235 (7)	
C32	0.3449 (5)	0.6269 (3)	0.7118 (3)	0.0311 (7)	
C33	0.4251 (6)	0.6979 (3)	0.7875 (3)	0.0379 (9)	
H33	0.3617	0.7194	0.8569	0.046*	
C34	0.5987 (6)	0.7368 (3)	0.7604 (4)	0.0415 (10)	
H34	0.6563	0.7848	0.8120	0.050*	
C35	0.6882 (6)	0.7064 (3)	0.6592 (4)	0.0362 (8)	
H35	0.8062	0.7345	0.6399	0.043*	
C36	0.6062 (5)	0.6344 (3)	0.5846 (3)	0.0295 (7)	
H36	0.6703	0.6138	0.5152	0.035*	

### Atomic displacement parameters (Å^2^)

**Table:**

	*U*^11^	*U*^22^	*U*^33^	*U*^12^	*U*^13^	*U*^23^
Br1	0.03347 (18)	0.0590 (2)	0.02960 (18)	0.0109 (2)	0.00755 (12)	0.0032 (2)
O1	0.0266 (11)	0.0314 (11)	0.0249 (12)	0.0089 (9)	0.0029 (9)	0.0033 (10)
C2	0.0192 (13)	0.0208 (17)	0.0281 (15)	0.0000 (12)	−0.0017 (11)	0.0022 (14)
C3	0.0187 (13)	0.0227 (19)	0.0279 (16)	0.0003 (11)	−0.0009 (12)	−0.0020 (12)
C4	0.0207 (14)	0.0209 (14)	0.0248 (16)	0.0016 (11)	−0.0025 (12)	−0.0020 (12)
C5	0.0245 (16)	0.0178 (14)	0.0296 (18)	0.0003 (12)	0.0014 (14)	−0.0013 (13)
C6	0.0309 (18)	0.0262 (17)	0.0315 (19)	0.0088 (14)	0.0010 (16)	−0.0020 (14)
C7	0.0290 (17)	0.0307 (18)	0.048 (2)	0.0116 (15)	0.0011 (16)	0.0000 (16)
C8	0.0317 (18)	0.0312 (18)	0.041 (2)	0.0104 (16)	−0.0047 (16)	0.0036 (16)
C9	0.0307 (17)	0.0264 (17)	0.0324 (19)	0.0023 (14)	−0.0041 (14)	0.0029 (14)
C10	0.0232 (15)	0.0189 (15)	0.0317 (18)	0.0020 (12)	−0.0009 (13)	0.0000 (13)
C11	0.0157 (13)	0.0228 (15)	0.0297 (18)	−0.0001 (12)	−0.0018 (12)	−0.0021 (13)
C12	0.0187 (14)	0.0226 (15)	0.0296 (17)	0.0011 (12)	−0.0008 (13)	−0.0019 (13)
C13	0.0204 (15)	0.0282 (16)	0.0218 (16)	0.0021 (12)	−0.0004 (12)	0.0000 (13)
C14	0.0187 (13)	0.030 (2)	0.0268 (15)	0.0019 (12)	−0.0004 (11)	−0.0008 (13)
N14	0.0192 (13)	0.0451 (18)	0.0375 (18)	−0.0102 (13)	−0.0045 (13)	0.0108 (15)
O141	0.0450 (16)	0.0380 (15)	0.0445 (18)	−0.0127 (13)	−0.0067 (15)	−0.0049 (13)
O142	0.077 (3)	0.105 (3)	0.060 (2)	−0.062 (2)	−0.013 (2)	0.030 (2)
C15	0.0223 (15)	0.0235 (16)	0.0324 (19)	0.0020 (12)	−0.0031 (13)	−0.0030 (14)
O15	0.0415 (14)	0.0308 (12)	0.0264 (14)	0.0070 (11)	0.0000 (11)	0.0040 (10)
C21	0.0254 (16)	0.0184 (15)	0.0260 (16)	0.0008 (12)	0.0010 (13)	0.0007 (13)
C22	0.0247 (16)	0.0304 (17)	0.0306 (18)	−0.0027 (13)	0.0010 (13)	−0.0031 (14)
C23	0.0259 (17)	0.0379 (19)	0.047 (2)	0.0003 (15)	0.0061 (16)	−0.0034 (17)
C24	0.0381 (19)	0.0295 (17)	0.035 (2)	0.0041 (15)	0.0103 (16)	−0.0060 (16)
C25	0.043 (2)	0.0305 (19)	0.0262 (18)	0.0017 (16)	−0.0006 (16)	−0.0034 (14)
C26	0.0260 (16)	0.0315 (18)	0.0273 (17)	0.0003 (14)	−0.0022 (13)	0.0011 (14)
C31	0.0249 (13)	0.0236 (19)	0.0219 (14)	0.0060 (12)	−0.0040 (11)	0.0024 (13)
C32	0.0339 (17)	0.0328 (18)	0.0266 (17)	0.0082 (15)	−0.0032 (14)	0.0022 (15)
C33	0.055 (2)	0.034 (2)	0.0239 (18)	0.0143 (18)	−0.0043 (16)	−0.0057 (15)
C34	0.055 (2)	0.0288 (18)	0.040 (2)	0.0041 (18)	−0.0176 (19)	−0.0057 (17)
C35	0.0363 (19)	0.0274 (17)	0.044 (2)	−0.0001 (15)	−0.0141 (17)	−0.0030 (16)
C36	0.0270 (16)	0.0294 (18)	0.0321 (19)	0.0024 (14)	−0.0041 (14)	−0.0004 (15)

### Geometric parameters (Å, °)

**Table:**

Br1—C32	1.902 (4)	C14—N14	1.534 (4)
O1—C2	1.379 (4)	C14—H14	1.0000
O1—C10	1.383 (4)	N14—O141	1.201 (4)
C2—C3	1.356 (4)	N14—O142	1.223 (5)
C2—C21	1.464 (4)	C15—O15	1.213 (4)
C3—C11	1.451 (4)	C15—H15	0.9500
C3—C4	1.514 (4)	C21—C22	1.398 (5)
C4—C14	1.515 (5)	C21—C26	1.401 (5)
C4—C5	1.520 (4)	C22—C23	1.377 (5)
C4—H4	1.0000	C22—H22	0.9500
C5—C10	1.382 (5)	C23—C24	1.395 (6)
C5—C6	1.398 (5)	C23—H23	0.9500
C6—C7	1.394 (5)	C24—C25	1.383 (5)
C6—H6	0.9500	C24—H24	0.9500
C7—C8	1.377 (6)	C25—C26	1.367 (5)
C7—H7	0.9500	C25—H25	0.9500
C8—C9	1.377 (5)	C26—H26	0.9500
C8—H8	0.9500	C31—C36	1.390 (5)
C9—C10	1.397 (5)	C31—C32	1.404 (5)
C9—H9	0.9500	C32—C33	1.387 (6)
C11—C12	1.345 (5)	C33—C34	1.383 (6)
C11—H11	0.9500	C33—H33	0.9500
C12—C15	1.465 (5)	C34—C35	1.373 (6)
C12—C13	1.511 (4)	C34—H34	0.9500
C13—C31	1.517 (4)	C35—C36	1.393 (5)
C13—C14	1.553 (4)	C35—H35	0.9500
C13—H13	1.0000	C36—H36	0.9500
			
C2—O1—C10	119.6 (3)	C4—C14—H14	108.4
C3—C2—O1	121.7 (3)	N14—C14—H14	108.4
C3—C2—C21	128.5 (3)	C13—C14—H14	108.4
O1—C2—C21	109.7 (3)	O141—N14—O142	122.1 (3)
C2—C3—C11	121.5 (3)	O141—N14—C14	120.3 (3)
C2—C3—C4	122.5 (3)	O142—N14—C14	117.5 (3)
C11—C3—C4	115.9 (3)	O15—C15—C12	124.6 (3)
C3—C4—C14	111.3 (3)	O15—C15—H15	117.7
C3—C4—C5	110.8 (3)	C12—C15—H15	117.7
C14—C4—C5	114.6 (3)	C22—C21—C26	118.8 (3)
C3—C4—H4	106.5	C22—C21—C2	122.2 (3)
C14—C4—H4	106.5	C26—C21—C2	119.0 (3)
C5—C4—H4	106.5	C23—C22—C21	120.1 (3)
C10—C5—C6	117.3 (3)	C23—C22—H22	120.0
C10—C5—C4	120.6 (3)	C21—C22—H22	120.0
C6—C5—C4	122.1 (3)	C22—C23—C24	120.5 (3)
C7—C6—C5	121.0 (4)	C22—C23—H23	119.7
C7—C6—H6	119.5	C24—C23—H23	119.7
C5—C6—H6	119.5	C25—C24—C23	119.3 (3)
C8—C7—C6	119.9 (3)	C25—C24—H24	120.3
C8—C7—H7	120.1	C23—C24—H24	120.3
C6—C7—H7	120.1	C26—C25—C24	120.6 (3)
C9—C8—C7	120.7 (3)	C26—C25—H25	119.7
C9—C8—H8	119.7	C24—C25—H25	119.7
C7—C8—H8	119.7	C25—C26—C21	120.7 (3)
C8—C9—C10	118.6 (3)	C25—C26—H26	119.7
C8—C9—H9	120.7	C21—C26—H26	119.7
C10—C9—H9	120.7	C36—C31—C32	116.0 (3)
C5—C10—O1	122.3 (3)	C36—C31—C13	121.8 (3)
C5—C10—C9	122.5 (3)	C32—C31—C13	122.1 (3)
O1—C10—C9	115.1 (3)	C33—C32—C31	122.9 (4)
C12—C11—C3	124.1 (3)	C33—C32—Br1	117.8 (3)
C12—C11—H11	117.9	C31—C32—Br1	119.3 (3)
C3—C11—H11	117.9	C34—C33—C32	118.9 (4)
C11—C12—C15	118.9 (3)	C34—C33—H33	120.6
C11—C12—C13	122.9 (3)	C32—C33—H33	120.6
C15—C12—C13	117.8 (3)	C35—C34—C33	120.2 (4)
C12—C13—C31	114.6 (3)	C35—C34—H34	119.9
C12—C13—C14	108.2 (3)	C33—C34—H34	119.9
C31—C13—C14	111.7 (3)	C34—C35—C36	120.1 (4)
C12—C13—H13	107.4	C34—C35—H35	119.9
C31—C13—H13	107.4	C36—C35—H35	119.9
C14—C13—H13	107.4	C31—C36—C35	121.9 (3)
C4—C14—N14	112.2 (3)	C31—C36—H36	119.1
C4—C14—C13	112.5 (3)	C35—C36—H36	119.1
N14—C14—C13	106.9 (3)		
			
C10—O1—C2—C3	−8.5 (4)	C5—C4—C14—C13	−176.7 (3)
C10—O1—C2—C21	169.5 (3)	C12—C13—C14—C4	−51.2 (3)
O1—C2—C3—C11	169.1 (3)	C31—C13—C14—C4	75.8 (3)
C21—C2—C3—C11	−8.6 (5)	C12—C13—C14—N14	72.3 (3)
O1—C2—C3—C4	−6.0 (5)	C31—C13—C14—N14	−160.7 (3)
C21—C2—C3—C4	176.3 (3)	C4—C14—N14—O141	0.6 (4)
C2—C3—C4—C14	143.9 (3)	C13—C14—N14—O141	−123.1 (3)
C11—C3—C4—C14	−31.4 (4)	C4—C14—N14—O142	177.4 (4)
C2—C3—C4—C5	15.1 (4)	C13—C14—N14—O142	53.7 (5)
C11—C3—C4—C5	−160.2 (3)	C11—C12—C15—O15	−173.6 (3)
C3—C4—C5—C10	−11.3 (4)	C13—C12—C15—O15	−0.6 (5)
C14—C4—C5—C10	−138.3 (3)	C3—C2—C21—C22	−43.0 (5)
C3—C4—C5—C6	169.7 (3)	O1—C2—C21—C22	139.1 (3)
C14—C4—C5—C6	42.7 (4)	C3—C2—C21—C26	139.9 (3)
C10—C5—C6—C7	1.7 (5)	O1—C2—C21—C26	−38.0 (4)
C4—C5—C6—C7	−179.2 (3)	C26—C21—C22—C23	−0.8 (5)
C5—C6—C7—C8	0.1 (6)	C2—C21—C22—C23	−177.9 (3)
C6—C7—C8—C9	−1.6 (6)	C21—C22—C23—C24	1.2 (6)
C7—C8—C9—C10	1.2 (6)	C22—C23—C24—C25	−1.0 (6)
C6—C5—C10—O1	177.6 (3)	C23—C24—C25—C26	0.4 (6)
C4—C5—C10—O1	−1.5 (5)	C24—C25—C26—C21	0.0 (5)
C6—C5—C10—C9	−2.2 (5)	C22—C21—C26—C25	0.2 (5)
C4—C5—C10—C9	178.7 (3)	C2—C21—C26—C25	177.4 (3)
C2—O1—C10—C5	12.3 (5)	C12—C13—C31—C36	19.3 (4)
C2—O1—C10—C9	−167.9 (3)	C14—C13—C31—C36	−104.2 (3)
C8—C9—C10—C5	0.8 (5)	C12—C13—C31—C32	−158.9 (3)
C8—C9—C10—O1	−179.0 (3)	C14—C13—C31—C32	77.7 (4)
C2—C3—C11—C12	−172.0 (3)	C36—C31—C32—C33	−1.8 (5)
C4—C3—C11—C12	3.3 (4)	C13—C31—C32—C33	176.5 (3)
C3—C11—C12—C15	172.8 (3)	C36—C31—C32—Br1	179.7 (2)
C3—C11—C12—C13	0.2 (5)	C13—C31—C32—Br1	−2.1 (4)
C11—C12—C13—C31	−101.9 (4)	C31—C32—C33—C34	0.8 (6)
C15—C12—C13—C31	85.4 (4)	Br1—C32—C33—C34	179.3 (3)
C11—C12—C13—C14	23.4 (4)	C32—C33—C34—C35	0.9 (6)
C15—C12—C13—C14	−149.3 (3)	C33—C34—C35—C36	−1.4 (6)
C3—C4—C14—N14	−64.0 (3)	C32—C31—C36—C35	1.2 (5)
C5—C4—C14—N14	62.7 (3)	C13—C31—C36—C35	−177.1 (3)
C3—C4—C14—C13	56.5 (3)	C34—C35—C36—C31	0.4 (5)

### Hydrogen-bond geometry (Å, °)

**Table:**

*D*—H···*A*	*D*—H	H···*A*	*D*···*A*	*D*—H···*A*
C4—H4···O15^i^	1.00	2.34	3.266 (4)	154.
C7—H7···O15^ii^	0.95	2.55	3.487 (5)	168.
C25—H25···O15^iii^	0.95	2.45	3.314 (4)	151.

Symmetry codes: (i) −*x*+1, *y*+1/2, −*z*+1; (ii) −*x*, *y*+1/2, −*z*+1; (iii) *x*, *y*, *z*−1.
